# Prediction model to identify patients with hypereosinophilic syndrome using real-world data

**DOI:** 10.1016/j.jacig.2025.100588

**Published:** 2025-10-24

**Authors:** Paneez Khoury, Yen Chung, Donna Carstens, Erin E. Cook, Fan Mu, Mu Cheng, Elizabeth Judson, Jingyi Chen, Travis Wang, Zhuo Chen, Princess U. Ogbogu

**Affiliations:** a National Institute of Allergy and Infectious Diseases, Bethesda, Md; b AstraZeneca Pharmaceuticals, Wilmington, Del; c Analysis Group Inc, Boston, Mass; d University Hospitals Rainbow Babies and Children’s Hospital, Cleveland, Ohio; e Case Western Reserve University School of Medicine, Cleveland, Ohio

**Keywords:** Hypereosinophilic syndrome, machine learning, prediction model, prevalence

## Abstract

**Background:**

Hypereosinophilic syndrome (HES) is challenging to diagnose and identify in real-world data.

**Objective:**

We sought to develop a machine learning model to predict HES diagnosis in secondary data and estimate HES prevalence among individuals with elevated blood eosinophil count (BEC).

**Methods:**

Open medical/pharmacy claims were used to develop a prediction model, including patients with ≥1 HES diagnosis code and ≥1 elevated BEC (>1,000 cells/μL), and non-HES controls with elevated BEC. Candidate predictors for HES diagnosis included demographics, disease manifestations, treatments, health care utilization, and procedures. A generalized linear mixed model with a binomial distribution and logit link function was used to construct the model. Model performance was evaluated using 5-fold cross-validation.

**Results:**

A total of 260 patients with a HES diagnosis and 157,718 non-HES controls with elevated BEC were included. Predictors with the largest coefficients included bone marrow biopsy, eosinophilic gastrointestinal disorders, eosinophilic granulomatosis with polyangiitis, and autoimmune disease of the digestive system. The final model achieved an area under the receiver operating characteristic curve of 0.82 and area under the precision recall curve of 0.83. After applying the model (0.7 predicted probability threshold), 6,233 patients with predicted HES were identified. These patients exhibited similar characteristics to patients with a HES diagnosis code. With this threshold, the prevalence of predicted HES and total HES (diagnosed plus predicted) was 5.30% and 5.65%, respectively, among those with an elevated BEC.

**Conclusion:**

A machine learning prediction model identified a substantial number of patients with predicted HES, suggesting that the actual prevalence of HES may be significantly underestimated.

Hypereosinophilic syndrome (HES) is a heterogeneous group of blood disorders characterized by eosinophil-mediated organ damage resulting from a persistently elevated blood eosinophil count (BEC) above 1,500 cells/μL.^1^ Symptoms of HES vary on the basis of the affected organs.^1^^,^^2^ The skin, respiratory tract, and gastrointestinal tract are most commonly affected, though HES can cause dysfunction in almost any organ system.^1^^,^^3^

Identification of patients with HES in both clinical practice and real-world data sources is challenging. The diagnosis and classification of HES in clinical practice is often complex and time-consuming because the heterogenous symptoms of HES necessitates a multidisciplinary approach and disease manifestations overlap with other eosinophilic disorders, such as eosinophilic granulomatosis with polyangiitis,^4^ eosinophilic gastrointestinal disorders,^5^ and myeloid neoplasms with eosinophilia.^6^^,^^7^ Moreover, there are currently no specialized assessment tools or reliable predictive biomarkers to identify disease activity.^7^

With regards to real-world evidence, identifying patients diagnosed with HES in secondary datasets is similarly difficult because HES was encoded under the general term *eosinophilia* until the introduction of the HES-specific code in October 2020. Therefore, identification of patients with HES in secondary databases, particularly those diagnosed before the introduction of the HES-specific International Classification of Disease (ICD) code, not only requires laboratory data on eosinophil counts but also the exclusion of other differential conditions through assessment of a combination of diagnoses, procedures, and treatments. As a rare disease, recognition of HES diagnosis solely on the basis of diagnostic codes to identify HES cases would likely result in an underestimated sample size. As a result, estimating population-based outcomes like prevalence and burden of HES require alternative methods to identify potentially undiagnosed cases within secondary databases.

Therefore, this study aimed to fill these gaps by first developing a prediction model using a machine learning approach to predict HES using administrative claims data and laboratory data among patients with elevated BEC (>1,000 cells/μL). Second, the model was then applied to compare patients with predicted versus diagnosed HES and to estimate the prevalence of HES among individuals with elevated BEC in the United States.

## Methods

### Data source

Study objectives were addressed using open medical and pharmacy claims from May 2017 to December 2021 supplied from the PatientSource database of Source Healthcare Analytics LLC, a Symphony Health Solutions Corporation, with a coverage of approximately 274 million patients annually throughout the United States. Approximately 70% of total patients had BEC data, which were pulled from May 2017 to October 2021. Additional details on the database are in the Methods section in this article’s Online Repository available at www.jaci-global.org.

The study was considered exempt research under 45 CFR § 46.104(d)(4) because it involved only the secondary use of data that were deidentified in compliance with the Health Insurance Portability and Accountability Act, specifically 45 CFR § 164.514.

### Prediction model

#### Model population

Patients were eligible for inclusion in the prediction model if they had ≥2 elevated BEC >1,000 cells/μL between May 2017 and October 2021. A threshold of >1,000 cells/μL was selected rather than a threshold of >1,500 cells/μL (the level typically used for a HES diagnosis) in order to capture a broader population of patients with HES, including prevalent patients who may have lower BEC due to treatment or other reasons. Elevated BECs not overlapping with any oral corticosteroid (OCS) use and not within 30 days of finishing a course of OCS were considered as candidate assessment dates for inclusion in the prediction model; patients were allowed to have more than one assessment date. The 30-day washout period was implemented to mitigate the lingering effects of OCS on BEC. The predictor assessment period required ≥12 months of continuous data activity (defined as consecutive encounters occurring <12 months apart) before and ≥6 months after the candidate assessment date to allow evaluation of selected predictors.

Patients were categorized into two cohorts according to the presence or absence of a HES diagnosis, as follows.

#### Patients with a HES diagnosis

Patients were required to have ≥1 claim with a HES diagnosis (ICD-10: D72.11x) after October 2020 and ≥1 elevated BEC after or within 6 months before the initial HES diagnosis. The first HES or eosinophilia diagnosis code (ICD-10: D72.10) in the data was considered to be the patient’s initial diagnosis. The eosinophilia diagnosis code was permitted because it may have been used for a diagnosis of HES before the introduction of the HES ICD-10 diagnosis code.

The following patients were excluded: patients with differential condition diagnosis codes (see Table E1 in the Online Repository available at www.jaci-global.org) associated with elevated BEC that were coded after the initial HES or eosinophilia diagnosis and who had an absence of further HES diagnosis codes occurring after the first differential condition. These patients were presumed to have alternative disease diagnoses.

#### Non-HES controls with elevated BEC

These control patients had no HES diagnosis code and had ≥6 months of continuous activity after October 2020 (to allow adequate observation time for a HES ICD-10 code after its introduction).

The model was trained and validated using all patients with a HES diagnosis and an equal-size, randomly selected set of non-HES controls with elevated BEC to mitigate class imbalance due to the rarity of HES.

#### Outcome measures

The outcome of the prediction model was a binary dependent variable (HES diagnosis: yes/no).

#### Candidate predictors

Candidate predictors were predefined variables based on clinical relevance to and testing for HES that were available in the dataset. The variable selection was guided by literature review and expert clinical input from specialists in HES. These comprised demographic characteristics at the assessment date and clinical features during the predictor assessment period, including BEC, conditions considered in the differential diagnosis of HES (differential conditions), disease manifestations, comorbidities, treatments, all-cause health care resource utilization (HRU), and diagnostic procedures. The full list of candidate predictors is provided in Table E1.

Candidate predictors were summarized descriptively for each assessment date contributed by patients with a HES diagnosis and non-HES controls with elevated BEC using means, standard deviations (SDs), and medians for continuous variables and counts and percentages for categorical variables.

#### Predictor selection and construction of the prediction model

Predictors of a HES diagnosis were selected using least absolute shrinkage and selection operator (LASSO) to avoid overfitting and reduce model complexity.^8^^,^^9^ Because each patient could contribute multiple assessment dates, a generalized linear mixed model with a binomial distribution and logit link function was used to construct the model using predictors selected by LASSO.^10^ Additional details on methodology can be found in the Methods section in the Online Repository.

#### Model prediction

The prediction model was then applied to all patients’ assessment dates to generate the predicted probability of being diagnosed with HES. If a patient had a predicted probability above a specified threshold for any assessment dates, the subject was categorized as a patient with predicted HES; otherwise, the subject was categorized as a non-HES control with elevated BEC. Various predictive thresholds, ranging from 0.1 to 0.9, were tested to determine the optimal threshold.

#### Evaluation of model performance

The model performance was evaluated using 5-fold cross-validation. Performance metrics such as sensitivity, specificity, positive predictive value (PPV), negative predictive value (NPV), area under the receiver operating characteristic curve (ROC AUC), and area under the precision recall curve (PR AUC)^11^, ^12^, ^13^ were calculated for each fold, then averaged across 5 folds (see Fig E1 in the Online Repository available at www.jaci-global.org). The optimal threshold was determined as the point where a balance between PPV and NPV was achieved. PPV and NPV were selected given the priority of understanding the clinical relevance of our algorithm and the known prevalence in our sample (randomly selected an equal-size set of non-HES controls). Further, because the prediction model aimed to identify undiagnosed HES, a higher PPV was desired over NPV to minimize the occurrence of false-positives.

### Characteristics of patients with diagnosed HES and predicted HES

The index date for the analysis of patient characteristics was defined as the first HES diagnosis (for diagnosed HES) or the first candidate assessment date with a predicted probability above the threshold of 0.7 (for predicted HES). Patient characteristics during the baseline period (ie, the 12 months before the index date) and treatment patterns, disease manifestations, and rates of HRU per person-year during the follow-up period (ie, the 6 months following the index date) were summarized and compared between patients with a HES diagnosis and patients with predicted HES.

### Statistical analysis

Means, SDs, and medians were reported for continuous variables, and counts and percentages were reported for categorical variables. Comparisons were made between patients with a HES diagnosis and patients with predicted HES by Wilcoxon rank sum test for continuous variables and chi-square or Fisher exact test for categorical variables. The methods used to assess the prevalence of HES are described in Methods section in the Online Repository.

## Results

### Prediction model

**Fig 1:**
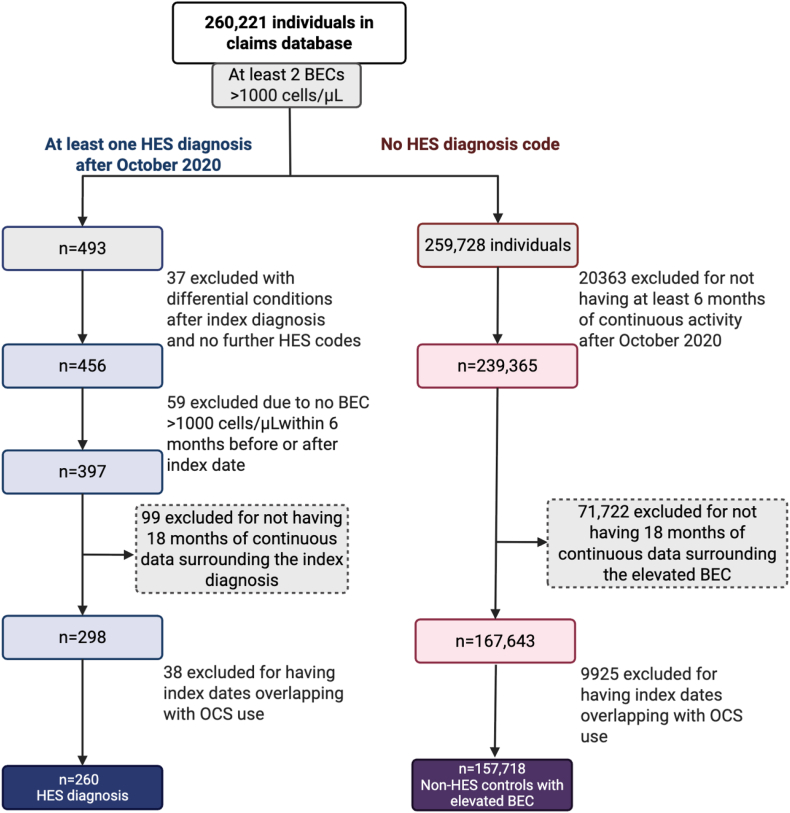
Sample selection. Created in BioRender.com. Khoury, P. (2025) https://BioRender.com/y23g3vl

A total of 260 patients with a HES diagnosis, contributing 661 candidate assessment dates, and 157,718 non-HES controls with elevated BEC, contributing 253,597 candidate assessment dates, were included for model development (Fig 1). Among patients with a HES diagnosis, the mean (SD) age was 55.7 (19.4) years across all assessment dates, and 54.3% were female. Among non-HES controls with elevated BEC, the mean (SD) age was 58.6 (19.4) years across all assessment dates, and 47.4% were female (Table I).

**Table I:** Patient demographics and clinical characteristics during the model evaluation period across all eligible assessment dates

Characteristic	HES diagnosis (n = 661)	Non-HES with elevated BEC (n = 253,597)
**Demographics at assessment dates**		
Age (years)	55.7 ± 19.4 [61.0]	58.6 ± 19.4 [64.0]
Sex		
Female	359 (54.3)	120,304 (47.4)
Male	302 (45.7)	133,293 (52.6)
Region		
Northeast	166 (25.1)	58,870 (23.2)
Midwest	179 (27.1)	78,375 (30.9)
Southeast	40 (6.1)	20,684 (8.2)
Southwest	112 (16.9)	42,334 (16.7)
West Coast	158 (23.9)	51,295 (20.2)
Unknown	6 (0.9)	2,039 (0.8)
Year of assessment date		
2018	91 (13.8)	60,569 (23.9)
2019	162 (24.5)	75,627 (29.8)
2020	235 (35.6)	78,509 (31.0)
2021	173 (26.2)	38,892 (15.3)
**Differential conditions**		
Allergic disease	314 (47.5)	60,487 (23.9)
Solid tumors	251 (38.0)	52,197 (20.6)
Autoimmune diseases	141 (21.3)	26,439 (10.4)
Immune deficiency/dysregulation	59 (8.9)	6,045 (2.4)
**Disease manifestations**		
Upper airway/pulmonary	399 (60.4)	94,716 (37.3)
Constitutional∗	315 (47.7)	78,705 (31.0)
Gastrointestinal	295 (44.6)	67,549 (26.6)
Dermatologic	256 (38.7)	53,434 (21.1)
Hematologic	209 (31.6)	61,408 (24.2)
Cardiovascular	152 (23.0)	36,542 (14.4)
Neurologic	169 (25.6)	41,755 (16.5)
**Other comorbidities and psychological conditions**		
Charlson comorbidity index	1.6 ± 1.9 [1.0]	1.3 ± 1.8 [1.0]
Anxiety disorders	132 (20.0)	30,677 (12.1)
Cognitive functioning	122 (18.5)	30,158 (11.9)
Sleep disorders	110 (16.6)	31,993 (12.6)
Depressive disorders	99 (15.0)	29,845 (11.8)
**Diagnostic procedures**		
Complete blood count with differential	397 (60.1)	77,058 (30.4)
CT scan (chest, pelvis, abdomen)	190 (28.7)	34,075 (13.4)
Lymphocyte phenotyping	171 (25.9)	5,669 (2.2)
Echocardiogram	150 (22.7)	30,897 (12.2)
Bone marrow biopsy	145 (21.9)	2,918 (1.2)
Troponin test	144 (21.8)	22,535 (8.9)
Genetic tests	91 (13.8)	3,885 (1.5)
Immunoassay	86 (13.0)	2,380 (0.9)
Liver panel	66 (10.0)	10,509 (4.1)
**BEC**		
BEC on assessment date (cells/μL)	3,140.5 ± 3,983.5 [1,832.0]	1,549.2 ± 1,069.0 [1,270.0]
>1,000-1,500 cells/μL	231 (34.9)	174,815 (68.9)
>1,500-3,000 cells/μL	265 (40.1)	67,673 (26.7)
>3,000 cells/μL	165 (25.0)	11,109 (4.4)
Highest BEC (cells/μL)	5,011.5 ± 6,355.9 [2,530.0]	1,792.8 ± 1,554.3 [1,398.0]
>1,000-1,500 cells/μL	105 (15.9)	145,580 (57.4)
>1,500-3,000 cells/μL	278 (42.1)	87,755 (34.6)
>3,000 cells/μL	278 (42.1)	20,262 (8.0)
**Treatment**		
Any corticosteroids	372 (56.3)	109,730 (43.3)
Corticosteroids (oral)	253 (38.3)	63,498 (25.0)
No. of days on OCS	54.0 ± 60.4 [30.0]	31.6 ± 51.3 [12.0]
Cumulative OCS dosage (mg)†	1,207.7 ± 1,636.6 [566.3]	721.6 ± 1,439.0 [300.0]
Daily average OCS dosage (mg)†	25.6 ± 25.8 [22.1]	27.3 ± 23.5 [21.0]
Corticosteroids (injection)	119 (18.0)	31,475 (12.4)
Corticosteroids (topical)	157 (23.8)	39,908 (15.7)
Corticosteroids (inhalation)	112 (16.9)	19,496 (7.7)
Immunomodulatory/monoclonal antibody	67 (10.1)	4,360 (1.7)
**HRU**		
Inpatient admission	188 (28.4)	63,206 (24.9)
No. of inpatient admissions	0.6 ± 1.4 [0.0]	0.6 ± 1.7 [0.0]
Length of inpatient stay among patients with ≥1 inpatient admission	11.6 ± 17.0 [5.0]	12.6 ± 27.8 [5.0]
Outpatient visits	653 (98.8)	214,852 (84.7)
No. of visits	17.8 ± 18.4 [12.0]	13.3 ± 30.9 [6.0]
Emergency department visits	247 (37.4)	65,839 (26.0)
No. of visits	0.9 ± 1.6 [0.0]	0.6 ± 1.8 [0.0]
Other visits	405 (61.3)	117,998 (46.5)
No. of visits	4.9 ± 9.6 [1.0]	3.6 ± 10.3 [0.0]

Values are reported as means ± SDs [medians] or nos. (%). Assessment dates were defined as any BEC > 1,000 cells/μL that were off OCS within preceding 30 days. Patients were allowed to contribute multiple assessment dates. Demographics were assessed at the assessment date; clinical characteristics were assessed during 1 year before the assessment date and 6 months after the assessment date. All patient characteristics were summarized at assessment date level. *CT,* Computed tomography.

∗ Group of symptoms or manifestations that indicate a systemic or general effect of a disease and that may affect general well-being or status of an individual (eg, muscle or joint pain, malaise, fatigue, fever).

† OCS dosage calculated based on its prednisone equivalent.

During the predictor assessment period, differential conditions were more common among patients with a HES diagnosis than non-HES controls with elevated BEC, including allergic diseases (47.5% [out of 661 assessment dates] vs 23.9% [out of 253,597 assessment dates], respectively), solid tumors (38.0% vs 20.6%), and autoimmune diseases (21.3% vs 10.4%). Similarly, disease manifestations were more common among patients with a HES diagnosis than non-HES controls with elevated BEC, including upper airway/pulmonary diseases (60.4% vs 37.3%), constitutional symptoms (47.7% vs 31.0%), and gastrointestinal disorders (44.6% vs 26.6%). A detailed breakdown of differential conditions and disease manifestations is reported in Table E2 in the Online Repository available at www.jaci-global.org.

Patients with a HES diagnosis had a higher median BEC at the assessment dates compared to non-HES controls with elevated BEC (1,832.0 vs 1,270.0 cells/μL).

The results of the best-fitting models are shown in Table II. The predictors with the largest coefficients included bone marrow biopsy, eosinophilic gastrointestinal disorders, eosinophilic granulomatosis with polyangiitis, and autoimmune disease of the digestive system; statistically significant predictors that were positively associated with having a HES diagnosis included record of bone marrow biopsy, higher BEC, and history of asthma.

**Table II:** Effect estimates of predictors selected by LASSO

Characteristic	Coefficient	Odds ratio	95% Confidence interval	*P* value
Bone marrow biopsy	6.06	430.20	3.92, 47218.28	.01∗
Eosinophilic gastrointestinal disorders	2.58	13.22	0.88, 199.34	.06
Eosinophilic granulomatosis with polyangiitis	2.55	12.78	0.02, 7662.50	.43
Autoimmune diseases—digestive system	2.23	9.26	0.56, 153.59	.12
BEC (reference >3,000 cells/μL)				
>1,000-1,500 cells/μL	−2.82	0.06	0.02, 0.20	<.001∗
>1,500-3,000 cells/μL	−1.34	0.26	0.08, 0.85	.03∗
Genetic tests	1.73	5.64	0.50, 63.78	.16
Urticaria/angioedema	1.50	4.49	0.38, 52.95	.23
Pruritis	1.44	4.21	0.71, 25.03	.11
Asthma	1.37	3.93	1.28, 12.12	.02∗
Immunoassay	1.21	3.36	0.35, 31.97	.29
Neoplasms of uncertain behavior, polycythemia vera and myelodysplastic syndromes	1.14	3.13	0.68, 14.45	.14
Immunomodulatory monoclonal antibody	1.09	2.96	0.28, 31.12	.37
Lymphocyte phenotyping	1.06	2.89	0.49, 17.05	.24
Malaise and fatigue	0.83	2.29	0.73, 7.12	.15
Rhinitis	0.74	2.09	0.65, 6.72	.21
Nasal polyposis	0.56	1.75	0.37, 8.19	.48
Abdominal pain	0.52	1.68	0.59, 4.83	.33
OCS duration (month)	0.13	1.13	0.76, 1.66	.52
Length of inpatient stay	−0.02	0.98	0.93, 1.04	.49
No. of inpatient visits	−0.09	0.91	0.61, 1.37	.67

**Fig 2:**
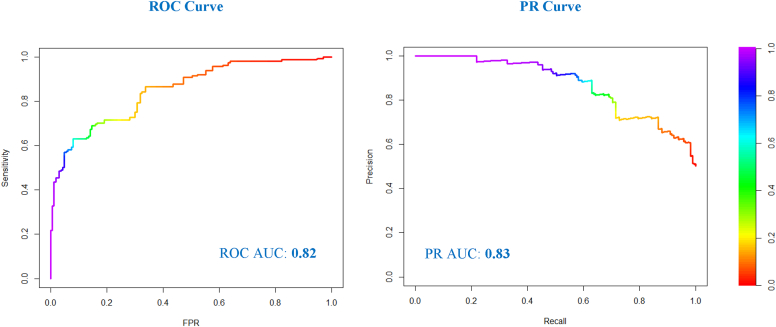
ROC AUC and PR AUC curves of final model. Curves were generated using one of the 5 folds of the validation results.

The final model achieved a ROC AUC of 0.82, with a PR AUC of 0.83 (Fig 2). Model performance statistics were evaluated using thresholds of predicted probability ranging from 0.1 to 0.9. PPV ranged from 0.654 to 0.928, and NPV ranged from 0.598 to 0.774. A threshold of 0.7 was selected because it offers a higher PPV while still maintaining a reasonable NPV (Table III).

**Table III:** Sensitivity, specificity, PPV, and NPV using different thresholds

Threshold	Sensitivity	Specificity	PPV	NPV
0.4	0.633	0.855	0.814	0.701
0.7	0.457	0.932	0.870	0.634
0.9	0.342	0.973	0.928	0.598

After applying the model to all eligible BECs among the 157,718 non-HES controls with elevated BEC in the model sample and using a 0.7 predicted probability threshold, a total of 6,233 patients with predicted HES were identified. The remaining 151,485 were classified as non-HES controls with elevated BEC.

### Characteristics of patients with diagnosed HES and predicted HES

The analysis of patient characteristics included 260 patients with a HES diagnosis and 6,233 patients with predicted HES. Overall, patients with predicted HES exhibited similar characteristics to patients with a HES diagnosis (Table IV). At baseline, patients with predicted and diagnosed HES had similar age (mean [SD]: 54.5 [20.7] vs 54.6 [19.9], *P* = .952) and sex distribution (female: 55.3% vs 52.7%, *P* = .438). Patients with predicted HES, compared to patients with a HES diagnosis, had higher prevalence of allergic diseases (55.5% vs 38.5%), solid tumors (33.1% vs 20.8%), and autoimmune diseases (22.6% vs 11.2%) as differential conditions, as well as a higher prevalence of disease manifestations, including upper airway/pulmonary (62.9% vs 47.7%), constitutional (42.5% vs 29.2%), and gastrointestinal (42.3% vs 30.4%, all *P* < .001). Patients with predicted HES had a lower median BEC at index than those with a HES diagnosis (1,653 vs 1,758 cells/μL, *P* < .001).

**Table IV:** Baseline patient characteristics for patients with a HES diagnosis and patients with predicted HES

Characteristic	HES diagnosis (n = 260)	Predicted HES (n = 6,233)	*P* value
Demographics			
Age (years)	54.6 ± 19.9 [59.0]	54.5 ± 20.7 [60.0]	.952
Sex			.438
Female	137 (52.7)	3,449 (55.3)	
Male	123 (47.3)	2,784 (44.7)	
Region			.078
Northeast	77 (29.6)	1,668 (26.8)	
Southeast	66 (25.4)	1,762 (28.3)	
West Coast	53 (20.4)	980 (15.7)	
Southwest	44 (16.9)	1,210 (19.4)	
Midwest	16 (6.2)	563 (9.0)	
Unknown	4 (1.5)	50 (0.8)	
Index year			<.001
2018	49 (18.8)	1,729 (27.7)	
2019	46 (17.7)	1,749 (28.1)	
2020	95 (36.5)	1,792 (28.8)	
2021	70 (26.9)	963 (15.5)	
**Differential conditions**			
Allergic disease	100 (38.5)	3,459 (55.5)	<.001
Solid tumors	54 (20.8)	2,065 (33.1)	<.001
Autoimmune diseases	29 (11.2)	1,410 (22.6)	<.001
Immune deficiency/dysregulation	13 (5.0)	365 (5.9)	.658
Hematological malignancy	11 (4.2)	680 (10.9)	<.001
Drug or toxin hypersensitivity	5 (1.9)	117 (1.9)	1.000
Adrenal insufficiency	3 (1.2)	49 (0.8)	.464
Parasitic infection	2 (0.8)	47 (0.8)	1.000
Viral infection	1 (0.4)	49 (0.8)	.723
Cholesterol embolization	1 (0.4)	2 (0.0)	.115
Bacterial infection	0	7 (0.1)	1.000
Systematic mastocytosis	0	4 (0.1)	1.000
Fungal infection	0	18 (0.3)	1.000
**Disease manifestations**			
Upper airway/pulmonary	124 (47.7)	3,919 (62.9)	<.001
Gastrointestinal	79 (30.4)	2,634 (42.3)	<.001
Constitutional∗	76 (29.2)	2,647 (42.5)	<.001
Dermatologic	65 (25.0)	2,421 (38.8)	<.001
Hematologic	45 (17.3)	1,931 (31.0)	<.001
Neurologic	34 (13.1)	1,100 (17.6)	.069
Cardiovascular	34 (13.1)	864 (13.9)	.789
Liver/spleen	14 (5.4)	327 (5.2)	1.000
Kidney	3 (1.2)	155 (2.5)	.217
Eosinophilic granulomatosis with polyangiitis	2 (0.8)	60 (1.0)	1.000
Patients with disease manifestation in >1 organ system	131 (50.4)	4,335 (69.5)	<.001
Patients with disease manifestation in >2 organ systems	73 (28.1)	2,968 (47.6)	<.001
**Comorbidities and psychological conditions**			
Charlson comorbidity index	0.9 ± 1.4 [0.0]	1.7 ± 1.9 [1.0]	<.001
Anxiety disorders	33 (12.7)	1,082 (17.4)	.061
Cognitive functioning	24 (9.2)	828 (13.3)	.071
Sleep disorders	26 (10.0)	986 (15.8)	<.05
Depressive disorders	26 (10.0)	931 (14.9)	<.05
**Diagnostic procedures**			
Complete blood count with differential	98 (37.7)	2,734 (43.9)	.057
CT scan (chest, pelvis, abdomen)	43 (16.5)	1,366 (21.9)	<.05
Echocardiogram	31 (11.9)	906 (14.5)	.278
Troponin test	27 (10.4)	720 (11.6)	.632
Lymphocyte phenotyping	21 (8.1)	735 (11.8)	.083
Bone marrow biopsy	11 (4.2)	658 (10.6)	<.01
Liver panel	16 (6.2)	396 (6.4)	1.000
**BEC**			
BEC on index date	3,111.5 ± 4,028.5 [1,757.5]	2,362.0 ± 2,736.9 [1,653.0]	<.001
>1,000-1,500 cells/μL	96 (36.9)	2,464 (39.5)	<.001
>1,500-5,000 cells/μL	126 (48.5)	3,408 (54.7)	
>5,000 cells/μL	38 (14.6)	361 (5.8)	
Highest BEC	4,249.1 ± 6,137.5 [2,143.0]	2,798.8 ± 3,243.1 [1,891.0]	<.001
>1,000-1,500 cells/μL	65 (25.0)	1,655 (26.6)	<.001
>1,500-5,000 cells/μL	136 (52.3)	4,023 (64.5)	
>5,000 cells/μL	59 (22.7)	555 (8.9)	

Values are reported as means ± SDs [medians] or nos. (%). Baseline demographics and differential conditions were summarized during 12-month preindex period. *CT,* Computed tomography.

∗ Group of symptoms or manifestations that indicate a systemic or general effect of disease and that may affect the general well-being or status of an individual (eg, muscle or joint pain, malaise, fatigue, fever).

During the 6-month follow-up period, similar treatment patterns were observed between the two cohorts. Similar proportions of patients with predicted HES and patients with a HES diagnosis used corticosteroids (46.6% vs 42.3%, *P* = .192). Mean (SD) OCS duration (predicted HES: 37.7 [40.6] vs HES diagnosis: 39.2 [40.9] days, *P* = .763) and cumulative OCS dosage (1,035.5 [1,642.6] vs 1,082.3 [1,829.1] mg prednisone equivalent, *P* = .82) were similar between the two cohorts (see Table E3 in the Online Repository available at www.jaci-global.org).

The proportions of disease manifestations during the follow-up period were generally similar between patients with predicted HES and those with a HES diagnosis. The most common disease manifestations were related to the upper airway/pulmonary (predicted HES: 53.8% vs HES diagnosis: 46.9%, *P* < .05), gastrointestinal (36.5% vs 29.6%, *P* < .05), and constitutional (35.9% vs 30.0%, *P* = .059) systems (see Table E4 in the Online Repository available at www.jaci-global.org).

Patients with predicted HES had comparable HRU during the follow-up period to patients with a HES diagnosis, including inpatient admissions (19.2% vs 17.7%, respectively, *P* = .590) and outpatient visits (92.6% vs 95.0%, *P* = .188; see Fig E2 in the Online Repository available at www.jaci-global.org). Emergency department visits were more common among patients with predicted HES than those with a HES diagnosis (24.7% vs 18.8%, *P* < .05).

### HES prevalence

**Fig 3:**
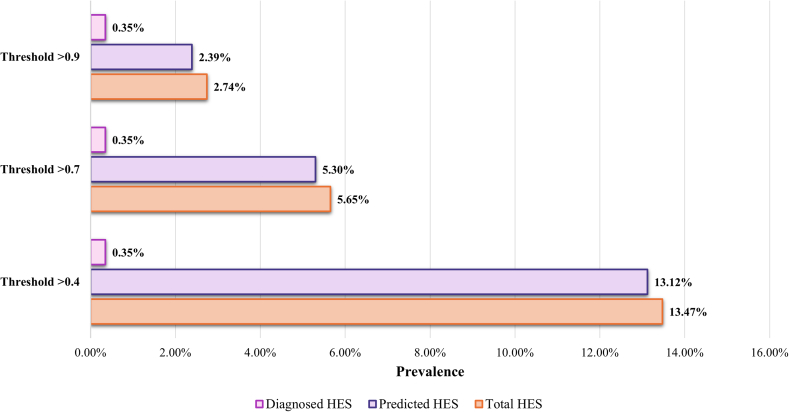
Prevalence of diagnosed HES, predicted HES, and total HES among patients with at least one BEC > 1,000 cells/μL from October 2020 to October 2021. Prevalence was calculated as the number of patients with a HES diagnosis, predicted HES, and total HES divided by the total number of patients with at least one assessment of BEC > 1,000 cells/μL from October 2020 to October 2021 and continuous data activity during this period.

During the period between October 2020 and October 2021, a total of 270 patients with a HES diagnosis were identified among 76,957 patients with elevated BEC, leading to a prevalence of 0.35% among individuals with an elevated BEC of >1,000/μL (Fig 3). With a predicted probability threshold of 0.7, the prevalence of predicted HES and total HES was 5.30% (n = 4,076) and 5.65% (n = 4,346), respectively, among those with an elevated BEC. With a threshold of 0.4, the estimated prevalence of predicted HES and total HES was 13.12% (n = 10,099) and 13.47% (n = 10,369), respectively. With the most stringent threshold of 0.9, the prevalence of predicted and total HES was 2.39% (n = 1,837) and 2.74% (n = 2,107).

## Discussion

This real-world study is the first to develop a United States claims-based prediction model using machine learning to predict diagnosis of HES among patients with elevated BEC. This model was then used to quantify the underdiagnosis and prevalence of HES in the United States. Among the 20 predictors included in the final model, bone marrow biopsy, higher BEC, and asthma had a significant positive association with HES diagnosis. Although none of the covariates can serve as a diagnostic criterion independently, each individual covariate has an impact on the probability of a patient’s being diagnosed with HES. Therefore, when considered collectively, these covariates emerge as valuable predictors of HES. In terms of model performance, the ROC AUC of 0.82 and PR AUC of 0.83 indicate a good ability to identify individuals with a high probability of having HES.^14^ The credibility of the model was further reinforced by the similar clinical profile observed between patients with predicted HES and those with a HES diagnosis, suggesting that the model effectively identified patients with characteristics consistent with the diagnosed HES cohort. As such, these patients with predicted HES may be representative of a larger, undiagnosed population with HES.

Recently, Requena et al^15^ developed a separate algorithm using a logistic regression model to identify patients with HES among a general population from electronic medical record data. The main predictors they identified included BEC ≥ 1,500 cells/μL during the 24 months before the index date (ie, date of HES diagnosis or qualifying BEC), potential HES treatment during the 6 months after index, and diagnosis of asthma, eosinophilia, or other white blood cell disorders. This model also demonstrated good performance, with a ROC AUC of 0.93. While the model building, predictor selection, and validation process were largely different from those of the current study, overlapping predictors were identified in the studies, including BEC, history of differential conditions or disease manifestations, and HES treatment receipt, which corroborate the findings of the present prediction model. In our study, we used LASSO for predictor selection, which offers the advantage of automatic variable selection and regularization to reduce overfitting and improve model interpretability. The consistency in key predictors across the two studies suggests the robustness of our findings despite differences in methodologic approaches.

To date, real-world prevalence of HES in the general population is not well characterized and is likely underestimated in the existing literature given the challenge of diagnosing HES in the context of other eosinophilic disorders with overlapping symptoms.^1^ One study conducted in the United States estimated a HES prevalence of 0.3 to 6.3 cases per 100,000 individuals between 2001 and 2005 as the product of HES incidence observed in Surveillance, Epidemiology, and End Results data multiplied by the median survival of HES patients.^16^ However, this prevalence estimate relied on many assumptions and is likely now outdated. A separate study in the United Kingdom estimated that the annual prevalence of HES ranged from 0.15 to 0.89 cases per 100,000 individuals between 2010 and 2018 using primary care data.^17^ In light of the limited epidemiology literature, the current study contributes an important, contemporary estimate of HES prevalence in the United States to fill this gap in knowledge.

The absence of an ICD code for HES before October 2020 has further complicated the evaluation of HES prevalence in real-world clinical practice. While a prevalence rate of 0.35% was estimated in this study on the basis of diagnosis codes alone, the prediction model used laboratory and administrative claims data to identify a substantial proportion of patients with undiagnosed HES, increasing the total estimated prevalence among patients with elevated BEC to 5.65% to 13.47% based on thresholds of 0.4 to 0.7. Even with the most conservative threshold (>0.9), the prevalence rate still exceeded 2%. Notably, the prediction model achieved a robust PPV, suggesting that patients with positive prediction model results are likely to have HES, strengthening the credibility of the prevalence estimates especially in light of similar treatments and OCS receipt applied to HES and predicted HES groups. As such, these findings highlight the underreporting of HES in the secondary data and the need for other tools like the present prediction model to comprehensively identify HES cases in these data sources.

Given the challenges of identifying HES in secondary data, there is limited real-world evidence characterizing the condition. The prediction model we have developed could, with further validation, be used with administrative claims data and other secondary databases to assess real-world clinical and economic outcomes, providing an algorithm-based solution to identify HES patients for further assessment. Indeed, prediction models that are based on claims data have commonly been used to assess developed and used to predict disease severity or prognosis for a variety of conditions,^18^^,^^19^, ^20^, ^21^ as well as used to detect rare diagnoses in claims databases.^22^ Validation of the current prediction model in independent datasets with access to clinical or laboratory data is warranted to ensure the model’s generalizability and accuracy. Findings from future real-world studies that use the validated prediction model may help us learn more about the burden, health impacts, and treatment decision-making process for patients with HES.

### Limitations

The study findings should be considered in the context of some limitations. Patients’ records were observable only if they had an encounter with a health system that reported their data to the open claims data source; therefore, encounters that occurred at medical centers outside of the contributing network were not visible. In addition, patients’ insurance enrollment information was not directly available, and data on race and ethnicity and other sociodemographics were limited, which restricted the ability to assess potential disparities in terms of access to tests, evaluations, and consultations. Similarly, some clinically relevant variables that may have been model predictors were not available in the data. For example, test results and imaging findings, such as from allergy tests and bone marrow biopsies, were not available in the structured data. As such, this prediction model cannot be used alone as a diagnostic tool for HES. Moreover, the defined predictors do not imply causal relationships or risk factors, and no individual diagnostic test should be interpreted as being predictive of a HES diagnosis. Instead, the prediction model may serve as a helpful tool to study HES retrospectively in secondary databases or to screen for a HES diagnosis in databases with subsequent adjudication.

Patients with true HES may have been misclassified and coded as non-HES, particularly around the time when the ICD-10 code was introduced (ie, October 2020), as not all providers or health systems may have been aware of the new code. Additionally, the model could not be used to extrapolate the prevalence of HES among the general population, as BEC were only available for a subset of the entire Symphony population, or to evaluate the incidence of HES given the left censoring of data, limiting the ability to accurately identify incident HES cases. Further, results are not generalizable to a population with a lower BEC. The requirement of having BEC > 1,000 cells/μL may have excluded some patients with HES diagnosed using a tissue eosinophil sample from this analysis.

The predicted outcome of the model and prevalence rates are subject to the threshold chosen. Choosing a cut point that increases the true positive rate would increase the false-positive rate (and vice versa). To improve the robustness of the model results, sensitivity analyses were conducted at various thresholds. Even with the most stringent threshold, the prevalence of total HES remained much higher than the prevalence based on HES diagnosis codes assigned, underscoring the difficulty of diagnosing HES and the likelihood of HES being underreported.

Last, the model was evaluated through a 5-fold cross-validation using the same dataset for model construction, as opposed to a separate holdout dataset, because of sample size limitations. Thus, enhancing the model in the future may involve incorporating more recent data and conducting external validation using a different secondary database for improved robustness.

### Conclusions

This real-world study presents the first prediction model developed for HES using US claims data, which may allow for identification of undiagnosed patients with HES and a more accurate estimate of the burden of HES in secondary data sources. The similarity in clinical profile between patients with a confirmed HES diagnosis and those with predicted HES highlights the comparable clinical burden and reinforces the credibility of the model. A substantial number of patients with predicted HES were identified by applying the prediction model, suggesting that the actual prevalence of HES may be significantly underestimated.

**Clinical implication**

This prediction model, when validated, may allow for the identification of undiagnosed patients with HES and a more accurate estimate of the burden of HES in secondary data sources.

## Disclosure statement

This study was funded by AstraZeneca Pharmaceuticals. This research was supported in part by the Intramural Research Program of the National Institutes of Health (NIH). The contributions of the NIH author (P.K.) were made as part of the author’s official duties as an NIH federal employee, are in compliance with agency policy requirements, and are considered works of the United States government. However, the findings and conclusions presented in this report are those of the authors and do not necessarily reflect the views of the NIH or the US Department of Health and Human Services.

Data availability statement: The data that support the findings of this study are available from Source Healthcare Analytics LLC, a Symphony Health Solutions Corporation. Restrictions apply to the availability of these data, which were used under license for this study. Data are available directly from Source Healthcare Analytics LLC, a Symphony Health Solutions Corporation.

Disclosure of potential conflict of interest: P. Khoury receives royalties from UptoDate and honoraria from Peerview LLC. Y. Chung, D. Carstens, and E. Judson are employees of AstraZeneca Pharmaceuticals. E. Cook, F. Mu, M. Cheng, J. Chen, T. Wang, and Z. Chen are employees of Analysis Group Inc, a consulting company that has provided paid consulting services to AstraZeneca Pharmaceuticals, which funded the development and conduct of this study and report. P. Ogbogu has received research support from AstraZeneca, GSK, Blueprint Medical, and DBV Technologies; has served as consultant for AstraZeneca; and has participated in advisory boards for Genentech and Novartis.
