# Supplementary material for: Prediction model to identify patients with hypereosinophilic syndrome using real-world data
Source: J Allergy Clin Immunol Glob. 2025 Oct 24;5(1):100588. doi: 10.1016/j.jacig.2025.100588 (PMC12651482; doi:10.1016/j.jacig.2025.100588)
Supplement: Supplementary Data [file mmc1.docx]

**Supplemental Materials: Prediction Model to Identify Patients with Hypereosinophilic Syndrome Using Real-World Data**

**Supplemental Methods**

**Data source**

The PatientSource® database of Source Healthcare Analytics, LLC, a Symphony Health Solutions Corporation includes healthcare information from a range of payers (e.g., Medicaid, Medicare, commercial insurance, and cash) on approximately 274 million patients annually throughout the United States (US). Data are sourced from pharmacy point-of-service sales, switch/network (i.e., clearing house) transactions, and additional direct prescriptions (e.g., medical and hospital claims data feeds), with approximately 90% of all retail prescription claims in the US included.

**Prediction model**

*Predictor selection*

Predictors of a hypereosinophilic syndrome (HES) diagnosis were selected using least absolute shrinkage and selection operator (LASSO) to avoid overfitting and reduce model complexity through L1 regularization to enhance robustness.^8,9^ By constructing a penalty function, LASSO shrinks regression coefficients and automatically performs variable selection by setting some coefficients to zero. The optimal penalty tuning parameter *λ* of the lasso was determined by minimizing Bayesian Information Criterion across a grid of 500 values.

*Construction of the prediction model*

Since each patient could contribute multiple assessment dates, a generalized linear mixed model (GLMM) with a binomial distribution and logit link function was used to construct the model using predictors selected by LASSO.^10^ GLMM was used to analyze repeated measurements on the same subject by incorporating a subject-specific random effect to the model to account for the intra-correlations of repeated measurements within each subject.

**Prevalence of HES and predicted HES among patients with elevated blood eosinophil count (BEC)**

*Prevalence population*

To be included as part of the prevalence analysis, patients were required to have ≥1 BEC >1,000 cells/µL and continuous data activity between October 2020 and October 2021.

Patient cohorts for the prevalence analysis were identified as follows:

- **Diagnosed HES**: patients with ≥1 HES diagnosis code between October 2020 and October 2021
- **Predicted HES**: patients without a HES diagnosis code but with a BEC >1,000 cells/µL at any time in the whole data period that met the probability thresholds to be classified as HES, as estimated from the prediction model. A minimum of 1 year of continuous data activity around the elevated BEC was required to ensure sufficient data for inclusion in the prediction model
- **Total HES**: both patients with a HES diagnosis and those with predicted HES.

The eligibility criteria applied for the prevalence analysis were broader than those of the model population to estimate the HES prevalence in a more generalizable population.

*Prevalence estimation*

Prevalence of diagnosed HES, predicted HES, and total HES were calculated as the number of patients in each group divided by the total number of patients with ≥1 BEC >1,000 cells/μL from October 2020 to October 2021.

**Supplementary Table 1. List of candidate predictors**

| **Candidate predictors** | **Type** | **ICD-10 code(s)** | **CPT/HCPCS code(s)** | **Time period of evaluation** |
| --- | --- | --- | --- | --- |
| ***Demographics*** |  |  |  |  |
| Age | Continuous | - | - | At assessment date |
| Sex | Binary | - | - |  |
| Geographic region | Categorical | - | - |  |
| ***Differential conditions*** |  |  |  |  |
| ***Allergic disease*** |  |  |  | 12-month pre- and 6-month post- assessment date |
| Allergic rhinitis | Binary | J30.1, J30.2, J30.3, J30.4 | - |  |
| Allergic asthma | Binary | J45 | - |  |
| Eczema/dermatitis | Binary | L20-L30 | - |  |
| Food allergy | Binary | J30.5, K52.21-K52.22, K52.29, L23.6, L24.6, L25.4, L27.2, T61.8-T61.9, T78.00-T78.09, T78.1, T78.2, Z91.010-T91.018 |  |  |
| Urticaria/angioedema | Binary | L50, T78.3 | - |  |
| ***Solid tumor*** |  |  |  |  |
| Benign neoplasms, except benign neuroendocrine tumors | Binary | D10-D36 | - |  |
| Neoplasms of uncertain behavior, polycythemia vera and myelodysplastic syndromes | Binary | D37-D48 | - |  |
| ***Autoimmune diseases*** |  |  |  |  |
| Digestive system | Binary | K900, K50, K51, K743 | - |  |
| Blood and immune system | Binary | D86, D510, D591, D693 | - |  |
| Nervous system | Binary | G35, G610, G700 | - |  |
| Eye and adnexa | Binary | H20 | - |  |
| Musculoskeletal system and connective tissue | Binary | M313, M05, M08, M33, M31, M353, M34, M32, M350, M45 | - |  |
| Skin and subcutaneous tissue | Binary | L12, L10, L400, L63, L80, L940 | - |  |
| ***Immune deficiency/dysregulation*** |  |  |  |  |
| Primary immunodeficiency | Binary | D80-D89 | - |  |
| ***Disease manifestations*** |  |  |  |  |
| ***Upper airway/pulmonary*** |  |  |  | 12-month pre- and  6-month post- assessment date |
| Asthma | Binary | J45 | - |  |
| Sinusitis | Binary | J01, J32 | - |  |
| Rhinitis | Binary | J30, J31.0 | - |  |
| Cough | Binary | R05 | - |  |
| Dyspnea | Binary | R06.0 | - |  |
| Shortness of breath | Binary | R06.02 | - |  |
| Wheezing | Binary | R06.2 | - |  |
| Pulmonary infiltrates | Binary | R91.8 | - |  |
| ***Constitutional*** |  |  |  |  |
| Fever | Binary | R50 | - |  |
| Weight loss | Binary | R63.4, R63.3, R62.51, P92, R62.7 | - |  |
| Malaise and fatigue | Binary | R53 | - |  |
| Muscle or join pain | Binary | M79.1, M25.5 | - |  |
| ***Dermatologic*** |  |  |  |  |
| Eczema | Binary | L20, L21, L2, L23-L25 | - |  |
| Pruritis | Binary | L29 | - |  |
| Purpura | Binary | D69 | - |  |
| Dermatitis | Binary | L20, L21, L23-L25, L30, L85.3, L41.3, L26, L27, B65.3, I87.2 | - |  |
| Unspecified edema | Binary | R60.9 | - |  |
| Rashes | Binary | R21 | - |  |
| ***Hematologic*** |  |  |  |  |
| Anemia | Binary | D50, D51, D52, D53, D55, D56, D57, D58, D5, D60, D61, D62, D63, D64 | - |  |
| Thrombocytopenia | Binary | D69.3, D69.4, D69.5, D69.6 | - |  |
| ***Gastrointestinal*** |  |  |  |  |
| Abdominal pain | Binary | R10 | - |  |
| Nausea/vomiting | Binary | R11 | - |  |
| Diarrhea | Binary | K59.1, R19.7 | - |  |
| Polyposis | Binary | K62.1, K63.5, K31.7, K51.4, D12, D13 | - |  |
| Ulceration | Binary | K22.1, K25, K26, K27, K28, K63.3 | - |  |
| EGIDs | Binary | K20.0, K52.81, K52.82 | - |  |
| ***Cardiovascular*** |  |  |  |  |
| Congestive heart failure | Binary | I09.9, I11.0, I13.0, I13.2, I25.5, I42.0, I42.5-I42.9, I43, I50, P29.0 | - |  |
| Valvular abnormality | Binary | A52.0, I05, I06, I07, I08, I09.1, I09.8, I34, I35, I36, I37, I38, I39, Q23.0, Q23.1, Q23.2, Q23.3, Z95.2, Z95.3, Z95.4 | - |  |
| ***Neurological*** |  |  |  |  |
| Peripheral neuropathy | Binary | G9050, G9051, G9052, G9059, G540, G55, G542, G544, E0841, E0941, E1041, E1141, E1341, G577, G59, G578, G588, G589, G64, G610, M055, E0840, E0842, E0940, E0942, E1040, E1042, E1140, E1142, E1340, E1342, G130, G131, A3683, A5215, G63, M3483, G621, G611, G620, G622, G6282, G6181, G6281, G6189, G6289, G619, G629, H463, M5410, M5418, M792, R20 | - |  |
| ***EGPA*** | Binary | M30.1 | - |  |
| ***HRU*** |  |  |  |  |
| Number of inpatient visits | Continuous | - | - | 12-month pre- and 6-month post- assessment date |
| Inpatient length of stay | Continuous | - | - |  |
| Number of outpatient visits | Continuous | - | - |  |
| Number of ED visits | Continuous | - | - |  |
| ***Treatment*** |  |  |  |  |
| ***Glucocorticoids*** | Binary |  |  | 12-month pre- and 6-month post- assessment date |
| Oral | Binary | - | - |  |
| OCS daily dosage | Continuous | - | - |  |
| OCS duration (month) | Continuous | - | - |  |
| OCS cumulative dosage | Continuous | - | - |  |
| Number of prescriptions | Continuous | - | - |  |
| Injection | Binary | - | - |  |
| Inhalation | Binary | - | - |  |
| Topical | Binary | - | - |  |
| ***Immunomodulatory monoclonal antibody*** | Binary | - | - |  |
| ***BEC*** |  |  |  |  |
| Highest BEC (>1000-1500, >1500-3000, >3000) | Categorical | - | - | 12-month pre- and 6-month post- assessment date |
| ***Key diagnostic procedures*** |  |  |  |  |
| CT scan (chest, pelvis, abdomen) | Binary | - | 71250, 71260, 71270, 71271, 72192, 72193, 72194, 74150, 74160, 74170, 74176, 74177, 74178 | 12-month pre- and 6-month post- assessment date |
| Echocardiogram | Binary | - | C8921, C8922, C8923, C8924, C8928, C8929, C8930, 93306, 93304, 93303, 93307, 93308, 93350, 93351, 93352,93320,93321, 93325 |  |
| Lymphocyte phenotyping | Binary | - | 86355, 86357, 86359, 86360 |  |
| Troponin test | Binary | - | 84484, 84512 |  |
| Bone marrow biopsy | Binary | - | G0364, 38221, 38220, 38222 |  |
| Immunoassay | Binary | - | 83520 |  |
| Genetic tests | Binary | - | 81105 -81364, 0001U-0241U, 81410-81471, 81479 |  |
| Liver panel | Binary | - | 80076 |  |
| ***Other comorbidities (CCI Score)*** |  |  |  |  |
| CCI Score | Continuous | - | - | 12-month pre- and 6-month post- assessment date |
| Myocardial infarction |  | I21.x, I22.x, I25.2 | - |  |
| Congestive heart failure |  | I09.9, I11.0, I13.0, I13.2, I25.5, I42.0, I42.5-I42.9, I43.x, I50.x, P29.0 | - |  |
| Peripheral vascular disorders |  | I70.x-I71.x, I73.1, I73.8-I73.9, I77.1, I79.0, I79.2, K55.1, K55.8-K55.9, Z95.8-Z95.9 | - |  |
| Cerebrovascular disease |  | G45.x-G46.x, H34.0, I60.x-I69.x | - |  |
| Dementia |  | F00.x-F03.x, F05.1, G30.x, G31.1 | - |  |
| Chronic pulmonary disease |  | I27.8-I27.9, J40.x-J47.x, J60.x-J67.x, J68.4, J70.1, J70.3 | - |  |
| Connective tissue disease |  | M05.x-M06.x, M31.5, M32.x-M34.x, M35.1, M35.3, M36.0 | - |  |
| Peptic ulcer disease |  | K25.x-K28.x | - |  |
| Mild liver disease |  | B18.x, K70.0-K70.3, K70.9, K71.3-K71.5, K71.7, K73.x-K74.x, K76.0, K76.2-K76.4, K76.8-K76.9, Z94.4 | - |  |
| Moderate or severe liver disease |  | I85.0, I85.9, I86.4, I98.2, K70.4, K71.1, K72.1, K72.9, K76.5-K76.7 | - |  |
| Diabetes with chronic complication |  | E10.2-E10.5, E10.7, E11.2-E11.5, E11.7, E12.2-E12.5, E12.7, E13.2-E13.5, E13.7, E14.2-E14.5, E14.7 | - |  |
| Diabetes without chronic complication |  | E10.0, E10.1x, E10.6, E10.8-E11.1, E11.6, E11.8, E11.9, E12.0, E12.1, E12.6, E12.8, E12.9, E13.0, E13.1, E13.6, E13.8, E13.9, E14.0, E14.1, E14.6, E14.8, E14.9 | - |  |
| Hemiplegia or paraplegia |  | G04.1, G11.4, G80.1-G80.2, G81.x-G82.x, G83.0-G83.4, G83.9 | - |  |
| Renal disease |  | I12.0, I13.1, N03.2-N03.7, N05.2-N05.7, N18.x-N19.x, N25.0, Z49.0-Z49.2, Z94.0, Z99.2 | - |  |
| Tumor without metastasis |  | K25.x-K28.x | - |  |
| Leukemia (acute or chronic) |  | C00.x-C26.x, C30.x-C34.x, C37.x-C41.x, C43.x, C45.x-C58.x, C60.x-C76.x, C97.x | - |  |
| Lymphoma |  | C91.x, C92.x, C93.x, C94.x, C95.x | - |  |
| Metastatic solid tumor |  | C81.x-C85.x, C88.x, C96.x | - |  |
| HIV/AIDS |  | C77.x-C80.x | - |  |
| ***HES flare*** |  |  |  |  |
| Number of flare episodes | Continuous | - | - | 6-month post- assessment date |

**Abbreviations**: BEC, blood eosinophil count; CCI, Charlson Comorbidity Index; CPT, Current Procedural Terminology; CT, computed tomography; ED, emergency department; EGIDs, eosinophilic gastrointestinal disorders; EGPA, eosinophilic granulomatosis with polyangiitis; HCPCS, Healthcare Common Procedure Coding System; HES, hypereosinophilic syndrome; HRU, healthcare resource use; ICD-10, International Classification of Diseases, tenth version; OCS, oral corticosteroid.

**Supplementary Table 2. Breakdown of differential conditions and disease manifestations during the model evaluation period across all eligible assessment dates^1,2^**

| ​ | **Assessment dates among patients with a HES** **diagnosis** **(N = 661**​) | **Assessment dates among non-HES controls with elevated BEC**  **(N = 253,597**​) |
| --- | --- | --- |
| **Differential conditions** | | |
| ***Allergic disease​*** | 314 (47.5%) | 60,487 (23.9%) |
| Allergic rhinitis | 88 (13.3%) | 10,626 (4.2%) |
| Allergic asthma | 183 (27.7%) | 31,786 (12.5%) |
| Eczema/dermatitis | 131 (19.8%) | 23,640 (9.3%) |
| Food allergy | 43 (6.5%) | 4,679 (1.8%) |
| Urticaria/angioedema | 42 (6.4%) | 3,306 (1.3%) |
| ***Solid tumors​*** | 251 (38.0%) | 52,197 (20.6%) |
| Benign neoplasms, except benign neuroendocrine tumors | 117 (17.7%) | 24,281 (9.6%) |
| Neoplasms of uncertain behavior, polycythemia vera and myelodysplastic syndromes | 133 (20.1%) | 16,044 (6.3%) |
| ***Autoimmune diseases​*** | 141 (21.3%) | 26,439 (10.4%) |
| Blood-forming organs and the immune mechanism | 13 (2.0%) | 2,370 (0.9%) |
| Nervous system | 20 (3.0%) | 2,216 (0.9%) |
| Eye and adnexa | 4 (0.6%) | 551 (0.2%) |
| Musculoskeletal system and connective tissue | 25 (3.8%) | 6,503 (2.6%) |
| Skin and subcutaneous tissue | 12 (1.8%) | 2,399 (0.9%) |
| ***Immune deficiency/dysregulation​*** | 59 (8.9%) | 6,045 (2.4%) |
| Primary immunodeficiency | 54 (8.2%) | 5,726 (2.3%) |
| **Disease manifestations** | | |
| ***Upper airway/pulmonary​*** | 399 (60.4%) | 94,716 (37.3%) |
| Asthma | 184 (27.8%) | 31,795 (12.5%) |
| Sinusitis | 85 (12.9%) | 15,858 (6.3%) |
| Rhinitis | 167 (25.3%) | 26,177 (10.3%) |
| Cough | 135 (20.4%) | 31,198 (12.3%) |
| Dyspnea | 177 (26.8%) | 38,074 (15.0%) |
| Shortness of breath | 154 (23.3%) | 30,998 (12.2%) |
| Wheezing | 60 (9.1%) | 5,332 (2.1%) |
| Pulmonary infiltrates | 103 (15.6%) | 15,425 (6.1%) |
| ***Constitutional*** | 315 (47.7%) | 78,705 (31.0%) |
| Fever | 48 (7.3%) | 15,634 (6.2%) |
| Weight loss | 40 (6.1%) | 8,233 (3.2%) |
| Malaise and fatigue | 161 (24.4%) | 34,342 (13.5%) |
| Muscle or join pain | 171 (25.9%) | 43,900 (17.3%) |
| ***Gastrointestinal​*** | 295 (44.6%) | 67,549 (26.6%) |
| Abdominal pain | 209 (31.6%) | 40,712 (16.1%) |
| Nausea/vomiting | 66 (10.0%) | 19,860 (7.8%) |
| Diarrhea | 66 (10.0%) | 14,899 (5.9%) |
| Polyposis | 84 (12.7%) | 14,376 (5.7%) |
| Ulceration | 34 (5.1%) | 5,525 (2.2%) |
| EGIDs | 54 (8.2%) | 1,453 (0.6%) |
| ***Dermatologic***​ | 256 (38.7%) | 53,434 (21.1%) |
| Eczema | 58 (8.8%) | 10,950 (4.3%) |
| Pruritus | 75 (11.3%) | 7,209 (2.8%) |
| Purpura | 58 (8.8%) | 7,934 (3.1%) |
| Dermatitis | 120 (18.2%) | 25,306 (10.0%) |
| Unspecified edema | 40 (6.1%) | 7,453 (2.9%) |
| Rashes | 93 (14.1%) | 9,724 (3.8%) |
| ***Hematologic​*** | 209 (31.6%) | 61,408 (24.2%) |
| Anemia | 181 (27.4%) | 57,298 (22.6%) |
| Thrombocytopenia | 39 (5.9%) | 6,325 (2.5%) |
| ***Cardiovascular*** | 152 (23.0%) | 36,542 (14.4%) |
| Congestive heart failure | 88 (13.3%) | 25,199 (9.9%) |
| Valvular abnormality | 90 (13.6%) | 18,723 (7.4%) |
| ***Neurological*** | 169 (25.6%) | 41,755 (16.5%) |
| Peripheral neuropathy | 127 (19.2%) | 31,329 (12.4%) |
| ***EGPA*** | 28 (4.2%) | 222 (0.1%) |

**Abbreviations:** BEC, blood eosinophil count; EGIDs, eosinophilic gastrointestinal disorders; EGPA, eosinophilic granulomatosis with polyangiitis; HES, hypereosinophilic syndrome; OCS, oral corticosteroid.

**Notes**:

[1] Assessment dates were defined as any BEC >1,000 cells/μL that were off OCS within the preceding 30 days. Patients were allowed to contribute multiple assessment dates.

[2] Clinical characteristics were assessed during the 1 year prior to and 6 months after the assessment date. All patient characteristics were summarized at the assessment date level.

**Supplementary Table 3. Treatment patterns during follow-up for patients with a HES diagnosis and patients with predicted HES^1^**

| ​ | **Patients with a HES diagnosis (N = 260)** | **Patients with predicted HES (N = 6,233)** | **P-value** |
| --- | --- | --- | --- |
| Any treatment | 122 (46.9%) | 3,312 (53.1%) | 0.057 |
| Any corticosteroids | 110 (42.3%) | 2,906 (46.6%) | 0.192 |
| OCS | 67 (25.8%) | 1,661 (26.6%) | 0.808 |
| Number of days on OCS | 39.2 ± 40.9 [21.0] | 37.7 ± 40.6 [19.0] | 0.763 |
| Cumulative OCS dosage (prednisone equivalent) (mg) | 1,082.3 ± 1,829.1 [490.0] | 1,035.5 ± 1,642.6 [450.0] | 0.823 |
| Topical corticosteroids | 36 (13.8%) | 811 (13.0%) | 0.766 |
| Immunomodulatory/monoclonal antibody | 21 (8.1%) | 599 (9.6%) | 0.474 |
| Cytotoxic agents^2^ | 6 (2.3%) | 210 (3.4%) | 0.448 |
| Immunosuppressive agents^3^ | 6 (2.3%) | 223 (3.6%) | 0.360 |
| TKI | 2 (0.8%) | 150 (2.4%) | 0.094 |
| JAK inhibitors | 0 (0.0%) | 22 (0.4%) | 1.000 |
| Interferon-alpha | 0 (0.0%) | 1 (0.0%) | 1.000 |
| Patients with more than 1 treatment category | 21 (8.1%) | 707 (11.3%) | 0.125 |

Values are reported as mean ± SD [median] or n (%).

**Abbreviations**: HES, hypereosinophilic syndrome; JAK, Janus kinase; OCS, oral corticosteroid; SD, standard deviation; TKI, tyrosine kinase inhibitor.

**Notes**:

[1] Follow-up period was defined as the 6-month post-index period.

[2] Cytotoxic agents included hydroxyurea, methotrexate, and vincristine.

[3] Immunosuppressive agents included azathioprine, busulfan, chlorambucil, cyclophosphamide, 2-chlorodeoxyadenosine alone or in combination with cytarabine, etoposide, mycophenolate mofetil, sirolimus, tacrolimus.

**Supplementary Table 4. Breakdown of disease manifestations during follow-up for patients with a HES diagnosis and patients with predicted HES^1^**

| ​ | **Patients with a HES diagnosis  (N = 260​)** | **Patients with predicted HES (N = 6,233​)** | **P-value** |
| --- | --- | --- | --- |
| **Upper airway/pulmonary** | 122 (46.9%) | 3,352 (53.8%) | < 0.05 |
| Asthma | 61 (23.5%) | 1,955 (31.4%) | < 0.01 |
| Dyspnea | 45 (17.3%) | 1,035 (16.6%) | 0.831 |
| Rhinitis | 44 (16.9%) | 1,345 (21.6%) | 0.086 |
| Shortness of breath | 38 (14.6%) | 805 (12.9%) | 0.481 |
| Cough | 34 (13.1%) | 879 (14.1%) | 0.708 |
| Pulmonary infiltrates | 21 (8.1%) | 469 (7.5%) | 0.833 |
| Sinusitis | 17 (6.5%) | 561 (9.0%) | 0.210 |
| Pleural effusion | 9 (3.5%) | 182 (2.9%) | 0.750 |
| Wheezing | 9 (3.5%) | 163 (2.6%) | 0.525 |
| Nasal polyps | 6 (2.3%) | 133 (2.1%) | 1.000 |
| Bronchiectasis | 5 (1.9%) | 101 (1.6%) | 0.898 |
| **Constitutional^2^** | 78 (30.0%) | 2,239 (35.9%) | 0.059 |
| Muscle or join pain | 44 (16.9%) | 916 (14.7%) | 0.367 |
| Malaise and fatigue | 40 (15.4%) | 1,258 (20.2%) | 0.069 |
| Fever | 9 (3.5%) | 394 (6.3%) | 0.082 |
| Weight loss | 11 (4.2%) | 334 (5.4%) | 0.514 |
| Night sweats | 1 (0.4%) | 49 (0.8%) | 0.723 |
| **Dermatologic** | 80 (30.8%) | 2,072 (33.2%) | 0.446 |
| Dermatitis | 34 (13.1%) | 931 (14.9%) | 0.461 |
| Rashes | 22 (8.5%) | 427 (6.9%) | 0.380 |
| Pruritus | 19 (7.3%) | 566 (9.1%) | 0.386 |
| Eczema | 16 (6.2%) | 484 (7.8%) | 0.403 |
| Purpura | 15 (5.8%) | 357 (5.7%) | 1.000 |
| Urticaria | 10 (3.8%) | 346 (5.6%) | 0.296 |
| Unspecified edema | 8 (3.1%) | 170 (2.7%) | 0.885 |
| Subcutaneous nodule | 7 (2.7%) | 197 (3.2%) | 0.808 |
| Mucosal erosions | 8 (3.1%) | 62 (1.0%) | < 0.01 |
| Angioedema | 3 (1.2%) | 55 (0.9%) | 0.504 |
| Erythroderma | 1 (0.4%) | 37 (0.6%) | 1.000 |
| Bullous lesions | 0 (0.0%) | 31 (0.5%) | 0.635 |
| Necrosis | 0 (0.0%) | 17 (0.3%) | 1.000 |
| Eosinophilic fasciitis | 0 (0.0%) | 5 (0.1%) | 1.000 |
| Eosinophilic cellulitis | 0 (0.0%) | 0 (0.0%) | - |
| **Hematologic** | 70 (26.9%) | 2,007 (32.2%) | 0.086 |
| Anemia | 59 (22.7%) | 1,815 (29.1%) | < 0.05 |
| Thrombocytopenia | 14 (5.4%) | 320 (5.1%) | 0.971 |
| Deep venous thrombosis | 7 (2.7%) | 136 (2.2%) | 0.739 |
| Myelofibrosis | 0 (0.0%) | 73 (1.2%) | 0.121 |
| Superficial thrombophlebitis | 0 (0.0%) | 8 (0.1%) | 1.000 |
| **Gastrointestinal** | 77 (29.6%) | 2,273 (36.5%) | < 0.05 |
| Abdominal pain | 45 (17.3%) | 1,440 (23.1%) | < 0.05 |
| Diarrhea | 18 (6.9%) | 600 (9.6%) | 0.178 |
| Nausea/vomiting | 18 (6.9%) | 597 (9.6%) | 0.185 |
| Polyposis | 15 (5.8%) | 447 (7.2%) | 0.460 |
| EGIDs | 15 (5.8%) | 328 (5.3%) | 0.829 |
| Ulceration | 8 (3.1%) | 151 (2.4%) | 0.643 |
| Strictures | 4 (1.5%) | 73 (1.2%) | 0.551 |
| Ascites | 2 (0.8%) | 65 (1.0%) | 1.000 |
| Cholecystitis | 0 (0.0%) | 25 (0.4%) | 0.624 |
| **Cardiovascular** | 48 (18.5%) | 792 (12.7%) | < 0.01 |
| Congestive heart failure | 26 (10.0%) | 504 (8.1%) | 0.323 |
| Valvular abnormality | 24 (9.2%) | 401 (6.4%) | 0.097 |
| Pericardial effusion | 4 (1.5%) | 61 (1.0%) | 0.332 |
| Cardiomyopathy | 1 (0.4%) | 45 (0.7%) | 1.000 |
| Myocarditis | 1 (0.4%) | 3 (0.0%) | 0.151 |
| Endomyocardial fibrosis | 0 (0.0%) | 3 (0.0%) | 1.000 |
| **Liver/spleen** | 9 (3.5%) | 341 (5.5%) | 0.206 |
| **Neurologic** | 37 (14.2%) | 889 (14.3%) | 1.000 |
| Peripheral neuropathy | 30 (11.5%) | 698 (11.2%) | 0.944 |
| Vertigo | 4 (1.5%) | 35 (0.6%) | 0.069 |
| Visual disturbances | 3 (1.2%) | 107 (1.7%) | 0.327 |
| Paresthesia | 3 (1.2%) | 74 (1.2%) | 1.000 |
| Hepatosplenomegaly | 2 (0.8%) | 185 (3.0%) | < 0.05 |
| Hepatomegaly | 2 (0.8%) | 106 (1.7%) | 0.327 |
| Mononeuritis multiplex | 2 (0.8%) | 8 (0.1%) | 0.058 |
| Change in cognition | 1 (0.4%) | 108 (1.7%) | 0.133 |
| Aphasia | 1 (0.4%) | 13 (0.2%) | 0.436 |
| **EGPA** | 8 (3.1%) | 86 (1.4%) | < 0.05 |
| **Kidney** | 6 (2.3%) | 151 (2.4%) | 1.000 |
| Proteinuria | 6 (2.3%) | 151 (2.4%) | 1.000 |
| **Patients with disease manifestations in more than 1 organ systems** | 145 (55.8%) | 4,051 (65.0%) | < 0.01 |
| **Patients with disease manifestations in more than 2 organ systems** | 83 (31.9%) | 2,500 (40.1%) | < 0.01 |

Values are reported as n (%).

**Abbreviations**: EGID, eosinophilic gastrointestinal disorder; EGPA, eosinophilic granulomatosis with polyangiitis; HES, hypereosinophilic syndrome.

**Notes:**

[1] Follow-up period was defined as the 6-month post-index period.

[2] Constitutional conditions referred to a group of symptoms or manifestations indicating a systemic or general effect of a disease and that may affect the general well-being or status of an individual (e.g., muscle or join pain, malaise and fatigue and fever).

**Supplementary Figure 1. Modeling procedure**


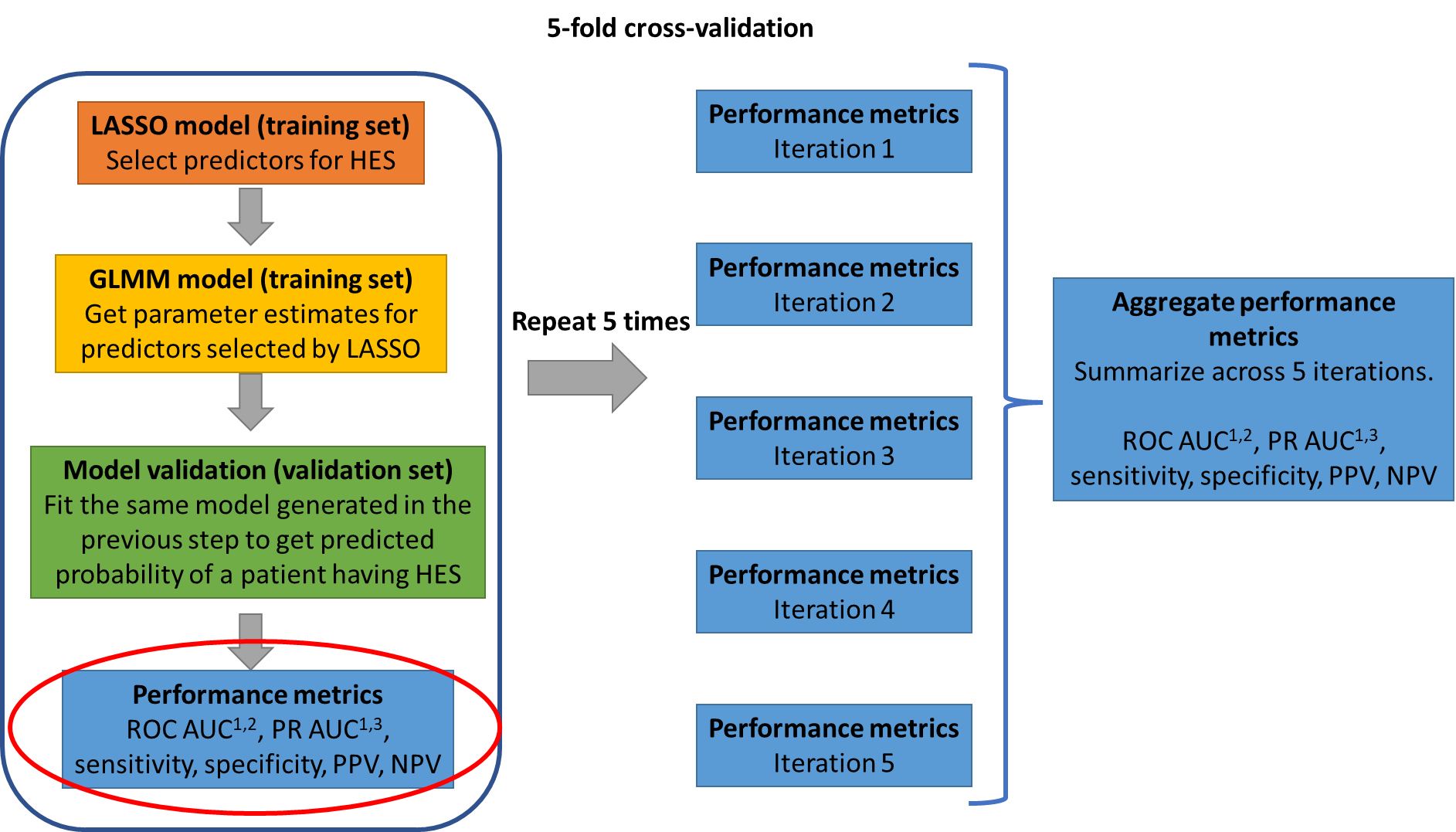


**Abbreviations**: AUC, area under the curve; GLMM, generalized linear mixed model; HES, hypereosinophilic syndrome; LASSO, least absolute shrinkage and selection operator; NPV, negative predictive value; PPV, positive predictive value; PR, precision recall; ROC, receiver operating characteristic.

**Notes:**

[1] The ROC AUC and PR AUC are both metrics used to evaluate the performance of binary classification models.

[2] The ROC curve plots true positive rate (sensitivity) vs. false positive rate (1 - specificity). A high ROC AUC suggests good overall discrimination ability of the model across different probability thresholds.

[3] The PR curve plots precision (positive predictive value) against recall (sensitivity). A high PR AUC indicates good model performance on identifying positive instances while minimizing false positives.

**Supplementary Figure 2. All-cause HRU during follow-up for patients with a HES diagnosis and patients with predicted HES^1^**

|  | **Patients with a HES diagnosis (N = 260)** | **Patients with predicted HES (N = 6,233)** | **P-value** |
| --- | --- | --- | --- |
| Patients with any inpatient admissions | 46 (17.7%) | 1,199 (19.2%) | 0.590 |
| Average length of stay per hospitalization (days) among patients with ≥1 inpatient admission | 4.6 ± 4.3 [3.3] | 4.5 ± 5.2 [3.0] | 0.920 |
| Patients with any outpatient visits | 247 (95.0%) | 5,774 (92.6%) | 0.188 |
| Patients with any ED visits | 49 (18.8%) | 1,541 (24.7%) | <0.05 |

Values are reported as mean ± SD [median] or n (%).

**Abbreviations**: ED, emergency department; HES, hypereosinophilic syndrome; HRU, healthcare resource utilization; SD, standard deviation.

**Note:**

[1] Follow-up period was defined as the 6-month post-index period.
